# In vitro fertilization outcome in frozen versus fresh embryo transfer in women with elevated progesterone level on the day of HCG injection: An RCT

**Published:** 2017-12

**Authors:** Marzieh Aghahosseini, Ashraf Aleyasin, Fatemeh Sadat Sarfjoo, Atossa Mahdavi, Mansooreh Yaraghi, Hojattollah Saeedabadi

**Affiliations:** 1 *Department of Obstetrics and Gynecology and Infertility, Shariati Hospital, Tehran University of Medical Sciences, Tehran, Iran.*; 2 *Department of Obstetrics, Gynecology and Pelvic Floor Disorders, Shariati Hospital, Tehran University of Medical Sciences, Tehran, Iran.*; 3 *Department of Embryology, Shariati Hospital, Tehran University of Medical Sciences, Tehran, Iran.*

**Keywords:** Progesterone, Embryo transfer, Pregnancy

## Abstract

**Background:**

The effect of elevated progesterone level on human chorionic gonadotropin (HCG) day in in vitro fertilization cycles is controversial. Some suppose that rise in progesterone level seems to have a negative impact on implantation and pregnancy by desynchronizing the endometrium, while others disagree.

**Objective:**

To evaluate the superiority of the frozen cycle over fresh cycle on live birth in patients with elevated progesterone level on HCG day.

**Materials and Methods:**

In this double-blind, randomized clinical trial, 72 women undergoing assisted reproductive technology with elevated progesterone level (≥1.8 ng/dl) on HCG day were included. The participants were grouped by fresh versus frozen embryo transfer, randomly. Finally, the clinical pregnancy and live birth rate were compared.

**Results:**

The implantation rate was 21.51%. The clinical pregnancy rate was 47.22% in fresh embryo transfer group (17/36) and 41.66% in frozen group (15/36) (p=0. 40). The live birth rate was not significantly difference between two groups (p=0.56).

**Conclusion:**

None of the fresh and frozen cycles are superior to the other and we recommend individualizing the decision for each patient. The frozen cycle may impose more emotional stress on patients.

## Introduction

The effect of elevated progesterone level on the day of human chorionic gonadotropin (HCG) injection on the possibility of pregnancy in in vitro fertilization (IVF) cycles was first noticed in 1991 (1). Many researchers analyzed its effect on the pregnancy rate in IVF cycles but contradictory results were achieved (2-4). One of its contradiction reasons is attributed to the selection of different thresholds for progesterone (0.9-3 ng/dl) in different studies. Another reason is the fact that most studies have been done retrospectively (5). Also, the effect of elevated progesterone level on the day of HCG injection on the pregnancy rate and the mechanism of this effect is unclear, but a premature rise in progesterone level seems to have a negative impact on implantation and pregnancy by desynchronizing the endometrium (6). Despite pituitary suppression by gonadotropin releasing hormones (GnRH) analogs, the prevalence of premature luteinizing hormone surge in assisted reproductive technology (ART) cycles is less than 2%; however, delayed progesterone elevation in the follicular phase that is caused by premature luteinizing hormone secretion, still occurs in 38% of IVF cycles. 

Although the primary role of progesterone is to support the luteal phase, fundamental research have shown a modest rise in the level of progesterone at the end of the follicular phase is due to physiological reasons and is associated with ovulation time and may even be essential for follicular maturation (7). In the latest meta-analysis conducted in 2013, several clinical questions remain unanswered in this context, these questions include the following: 1) what is the incidence of elevated progesterone level? 2) Does the progesterone elevation depend on ovarian response (low, moderate, high)? 3) Does the progesterone elevation on the day of HCG injection in the fresh cycles reduce the pregnancy rate as compared to frozen cycles (8)?

Considering the contradictory results obtained from published literature and the importance of pregnancy rates of IVF cycles, we decided to compare pregnancy rates in fresh and frozen cycles in women with elevated progesterone level in a prospective randomized study.

## Materials and methods

Infertile women, who were candidated for ART referring to Omid Fertility Center and Shariati Hospital from January to April 2016 enrolled in this double-blind, randomized clinical trial. A demographic form of medical records was filled for each participant.

Exclusion criteria were: uterine anomaly or previous uterine surgery, oocyte donation, azoospermia, severe endometriosis, previous chemotherapy or radiotherapy, conditions affecting the reproductive status. 

After an ultrasound examination in order to ensure a thin endometrium, lack of ovarian cysts, and antral follicle count analysis, gonadotropin administration on the 2^nd^ or 3^rd^ day of menstrual cycle was started. When the follicular size reached to 14 mm, GnRH antagonist, Cetrorelix (Cetrotide, MERCK, Germany) 250 µg/ day was prescribed.

After detection of three or more follicles measuring 17 mm, 10,000 units HCG (Pregnyl, MERCK, Germany) was injected intramuscularly and ultrasound guided oocyte retrieval was performed 36 hr later. In all participants blood progesterone level was measured using Vidas method (Automated Immunoanalyser BIOMERIEUX, France) before HCG injection. 

Women with a progesterone level ≥1.8 ng/dl were randomly divided into two groups according to the random allocation software. The transfer was conducted in the fresh and frozen cycles in group A and B, respectively. In group A, a total of 1 or 2 blastocysts (grade A) were transferred at 5^th^ day by two infertility clinicians. Luteal phase support was carried out for all participants by 100 mg Endometrin vaginal suppositories (FERRING, Switzerland) twice a day plus daily intramuscular injection of 50 mg of progesterone (Aboreihan, Iran). In group B, all embryos were cryopreserved by vitrification and after two menstrual cycles, endometrial preparation was performed. Estradiol valerate 6 mg/ day was begun from day three of menstrual cycle till the endometrial line of 8 mm. Then, 100 mg Endometrin vaginal suppositories was prescribed twice a day. A total of 1 or 2 grade A thawed blastocysts were transferred by two infertility clinicians after 5 days of progestin administration. Progestrone injection was the same as group A. 

Chemical pregnancy was defined as a positive βhCG 16 days after the embryo transfer, clinical pregnancy was described a gestational sac with a live fetus on ultrasound 5 wk after transfer and pregnancies with a gestational age greater than 24 wk was considered as a live birth. 

**Ethical consideration**

The protocol was approved by the Tehran University of Medical Sciences Ethics Committee (IR.TUMS.MEDICINE.REC.1395. 1251). Informed consent has been achieved from all participants. 

**Statistical analysis**

The main outcome of the study was the clinical pregnancy based on the reference that pregnancy rate in fresh and frozen groups were 10% and 47%, respectively (9).According to that, the sample size was estimated 36 individuals in each group, after taking into account a=0.05 and power of 95%. Chi-Square or Fisher Test, Independent *t*-test and one-way ANOVA were used to show independence of grouped variables, to compare the mean between the two independent groups and to compare between several independent groups, respectively. The relationship between the pregnancy outcome and effective variables was calculated using multivariate logistic regression with adjustment for confounding variables. All the analyses were carried out using STATA software v.13 and p≤0.05 was considered as statistically significant level.

## Results

**Figure 1:**
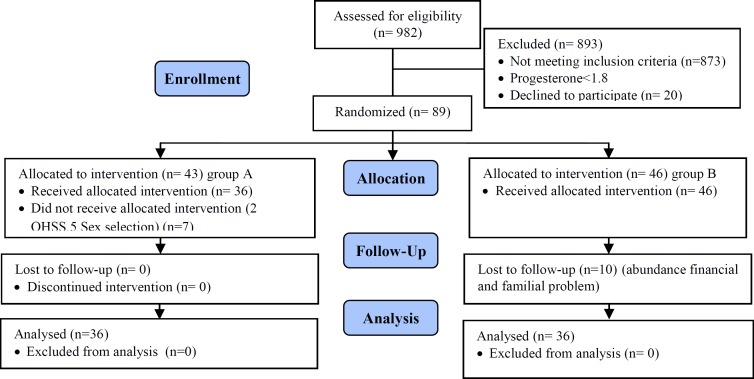
Algorithm for patients and outcomes.

In this study, 982 patients with ART cycles were assessed for the eligibility criteria. In these cycles, the progesterone level was measured on the HCG day and 89 cycles had a progesterone level of ≥1.8 ng/ dl with the incidence rate of 9%. A total of 17 women decided to withdraw from the treatment process and finally, 72 women were enrolled in the study (n=36/group) (Figure 1). 

There were no statistically significant differences in the demographic characteristics (age, BMI, and AMH) of both study groups (Table I).

The number of chemical pregnancy like clinical pregnancies in B and A was 15 (41.66%) and 17 cases (47.22%), respectively. The number of live birth in both groups was 15 cases (41.66%). Univariate analysis with the use of chi-square test demonstrates that there is no statistically significant difference between pregnancies and live birth rate in both B and A group (p=0.4, p=0.54) (Table II)., although 2 abortions occurred in fresh cycles, it was not statistically significant (p=0.3). All pregnancies were a singleton.

**Figure 2:**
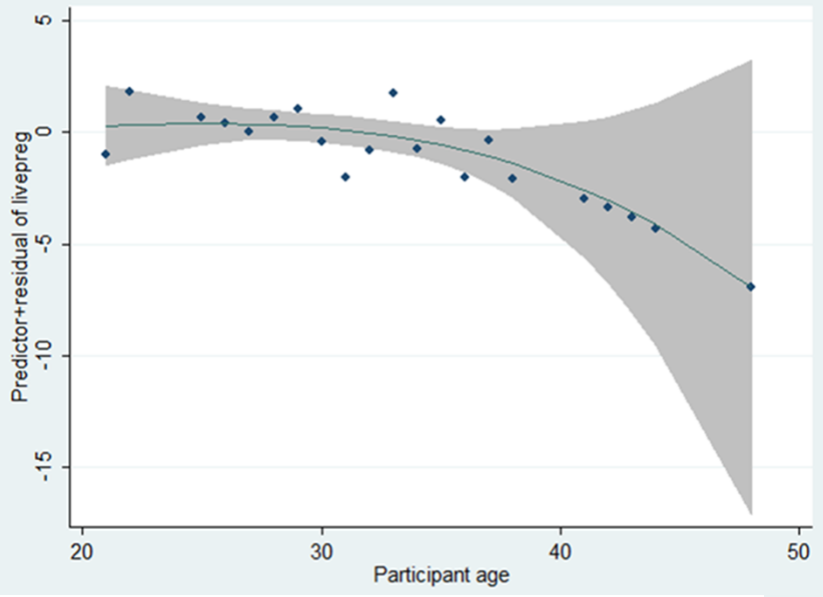
The relationship between age and the chance of pregnancy in women undergoing ART.

The results of multivariate logistic regression analysis showed that the age was the only variable affecting the occurrence of live birth pregnancy (Table III), so with 1 yr increase in age, the chance of pregnancy is significantly reduced by 16% from age 30 onwards (Figure 2). Chi-square analysis showed that there was no a significant relationship between these two variables in terms of the relationship between ovarian response and progesterone level≥1.8 and live birth pregnancy (p=0.54). According to the results, 2 cases (2.78%) had less than 4 eggs, 28 cases (38.89%) had 4-15 eggs, and 42 cases (58.33%) had more than 15 eggs. They were defined as poor, normal, and high ovarian response, respectively

**Table I:** Demographic characteristics of participants (n=36/group)

**Variables**	**Group A**	**Group B**	**Total**	**p-value**
Age (yr)	32.8 ± 5.8	30.5 ± 4.7	31.7 ± 5.4	0.66
BMI (kg/m^2^)	26 ± 4.72	26.2 ± 5.3	26.1 ± 4.98	0.82
AMH (ng/ml)	3.99 ± 0.36	4.99 ± 0.5	4.49 ± 2.68	0.11
Progesterone level (ng/ dl) on HCG day	2.59 ± 1	2.82 ± 1.38	2.71 ± 1.22	0.43
Duration of infertility (yr)	4.96 ± 4.44	5.4 ± 4	5.16 ± 4.19	0.65
Oocyte number	12 ± 6.5	19.6 ± 9.9	15.9 ± 9.15	0.0003
Metaphase II oocytes number	7.8 ± 5.1	12.8 ± 7.1	10.36 ± 6.6	0.001

Values are presented as mean ± SD. Independent *t*-test (n=36)

BMI: Body mass index.

AMH: Anti mullerian hormone.

HCG: Human chorionic gonadotropin

**Table II:** Pregnancy outcomes

**Variables**	**Group A (n= 36)**	**Group B (n= 36)**	p-value*
Chemical pregnancy	17 (47.22)	15 (41.66)	0.4
Clinical pregnancy	17 (47.22)	15 (41.66)	0.4
Live birth rate	15 (41.66)	15 (41.66)	0.54
Abortion rate	2(5)	0	0.3

* Chi-square test

**Table III:** Results of multivariate logistic regression analysis on the variables affecting pregnancy

**Variable **	**Category**	**OR, 95%CI**	**p-value**
Treatment group	Fresh	Reference	-
	Freeze	0.69 (0.19-2.44)	0.56
Age	yr	0.84 (0.73-0.97)	0.025
Total number of oocytes	number	0.95 (0.81-1.12)	0.6
MII (oocytes in metaphase II)	number	1.01 (0.83-1.24)	0.85
Progesterone level	ng/dl	1.44 (0.88-2.23)	0.13
AMH	ng/ml	0.97 (0.74-1.27)	0.85
Duration of infertility	yr	0.92 (0.79-1.08)	0.34

## Discussion

Our study revealed that there is no significant difference in the clinical pregnancy and live birth in patients with high progesterone level in both fresh and frozen cycles. Mechanism of action of elevated progesterone level on pregnancy outcomes in ART cycles is still controversial (2-4). A recent systematic meta-analysis conducted on 60,000 ART cycles in 2016, suggested the probability of the significant decrease in the pregnancy outcome in patients with elevated progesterone level in ART cycles by taking GnRH agonists and gonadotropins on the day of HCG injection (8). Various studies referred to very different threshold levels of 0.9-3 ng/ml as the negative effects of elevated progesterone level in assisted reproductive cycles (10). 

Park *and co-workers* in 2015 examined the factors contributing to the increase in progesterone level on the day of HCG injection. These factors were as the following: level of estradiol on the day of HCG injection, number of follicles ≤14 mm, number of oocytes obtained by a puncture and the ovary sensitivity. They stated that level of basic follicle-stimulating hormone; estradiol, age, body mass index and type of treatment protocol are ineffective (11). In our study, there was no significant difference in the clinical pregnancy and live birth in patients with high progesterone level (≤1.8) between fresh and frozen cycles. Most studies that support the negative effects of progesterone in ART cycles were retrospective studies. In a study by Martinez Francisca *and *colleagues (2009-2014) on 1896 ART cycles, no relationship has been found between elevated progesterone levels on the day of HCG injection and the clinical pregnancy. This study was also conducted prospectively (5). 

Another prospective study that examined the effects of progesterone and progesterone to estradiol ratio (P/E2) on the pregnancy rate was conducted by Shalom-Paz *et al* (12). In this study, P/E2 of less than 0.45 was associated with an increase in the chance of live birth pregnancy and elevated progesterone level alone had no effect on the pregnancy rate. They stated that successful implantation depends on estradiol level, endometrial thickness and synchronization of the embryonic stage, decidualization and endometrial receptivity and also expressed that in patients with excessive ovarian response, elevated progesterone level is associated with elevated estradiol level that prevents the negative effects of elevated progesterone level on implantation, thus it has no negative effect on live birth pregnancy. Perhaps the negative effects of high levels of progesterone and P/E2 ratio are somewhat modified 5 days after the transfer (12).

Highly variable thresholds that have been defined for high levels of progesterone and its negative effects could indicate that the negative effects of this hormone surge may depend on other factors and measuring progesterone alone is not a good predictive factor. In our study, most of the cycles had normal and above normal ovarian response (only two cycles had the poor ovarian response), which supports the hypothesis that elevated progesterone level in patients with poor ovarian response may be raised as a bad predictive factor and not in all patients. 

However, considering the low number of poor ovarian response, the results of our study are not generalizable to this group of patients. In our study, the pregnancy rate was equal in both groups. Although, frozen cycles were not affected by the progesterone levels. The only difference was observed in the abortion rate, which was zero in the frozen group, although this value was not significant statistically. 

There was a significant difference between the number of oocytes and mature oocytes between two groups, but they could not confound the final results. Because of similar basic characteristics like age, body mass index, AMH level in two comparison groups and computerized random allocation the results can be considered valuable for interpretation. Even similar pregnancy rates can support this evidence. 

In line with the results of our study, Ghasemi-Nejad and co-workers who conducted a study on high levels of progesterone and the pregnancy rate among Iranian patients with tubal infertility and PCO showed that elevated progesterone level also had no effect on pregnancy rate, which can be indicative of other factors involved in the interpretation of the elevated progesterone level (13). 

## Conclusion

According to our study, it seems that in patients with appropriate pregnancy conditions (good prognosis), it's better not to cancel the cycle simply because of the elevated progesterone level. The physician should decide for each patient considering overall conditions, because the frozen cycle may impose a more financial burden, increased stress and psychological pressure among couples

## Note

***Registration ID in IRCT: IRCT2016122131508N1***
